# Owner-Reported Cohort Study of Causes, Management and Outcome of Traumatic Wounds in 219 Horses

**DOI:** 10.3390/ani16101474

**Published:** 2026-05-11

**Authors:** Richard Birnie, Emmeline Hannelly, Julia Dubuc, Katie Burrell, Gary C. W. England, John H. Burford, Sarah L. Freeman

**Affiliations:** 1 Sydney Equine Practice, 8/19 Lyell Street, Mittagong, NSW 2575, Australia; richardbirnie97@gmail.com; 2 British Horse Society, Abbey Park, Stareton, Kenilworth CV8 2XZ, UK; emmeline.hannelly@bhs.org.uk; 3 Oakham Veterinary Hospital, Ashwell Road, Oakham LE15 7QH, UK; julia.dubuc@oakhamvethospital.co.uk; 4 School of Veterinary Medicine and Science, University of Nottingham, College Road, Sutton Bonington, Loughborough LE12 5RD, UK; katie.burrell1@nottingham.ac.uk (K.B.); gary.england@nottingham.ac.uk (G.C.W.E.); john.burford@nottingham.ac.uk (J.H.B.)

**Keywords:** equine, wound, owner, cause, first aid

## Abstract

Current evidence on traumatic equine wounds are predominantly from expert opinion reviews or referral hospital studies. This study aimed to describe the owner-reported causes, management and outcome of equine wounds. An owner-reported cohort approach was used. Owners of horses with recent traumatic wounds were recruited through snowball sampling. Data were collected from initial injury to final healing and outcome, including horse demographics, wound cause and location, owner- and veterinary-administered treatment, and outcome. Descriptive statistics included frequency percentages (%, x/y) and median and interquartile range. Data were obtained for 219 cases, with outcome data for 139 horses. The most common wound cause was a wire/fence injury (38%, 84/219), and the most common location was distal hindlimb (31%, 79/251). Owners administered initial first aid in 67% (147/219) of cases, and 75% (165/219) of horses received veterinary treatment. A total of 19% (38/201) of owners were not confident in deciding whether veterinary attention was needed. Wound healing time was a median of 60 days (IQR 30.3–157.0), time to return to work was a median of 6.5 weeks (IQR 2.0–16.0), and 3/139 horses were euthanised. Main study limitations were small sample size, self-selection, owner-reporting and attrition bias. Key areas for future resources to support owner preventative care and decision-making were identified.

## Simple Summary

Wounds are a common emergency problem in horses. The study asked horse owners to report any wound injuries in their horse, including what caused it, where the wound was, what first aid they gave, whether the horse had any veterinary treatment and what the outcome was. Owners sent in information for 219 horses with wound injuries, with final outcomes for 139 horses. The most common reason for wounds were wire/fencing injuries. The most common place that injuries occurred were on the lower half of the hindlegs. Owners gave first aid in just over two-thirds of cases, and three-quarters of horses needed veterinary attention. Owners were not always confident in deciding if the wound needed veterinary attention. The amount of time that it took for wounds to heal and for the horses to return to work varied greatly, but the average was 60 days to heal and six and half weeks to return to work. The study has shown that we need more research on wounds in the general horse population, not just those treated at referral hospitals. It has also identified areas where owners would benefit from support to help them with first aid and deciding when to call the vet.

## 1. Introduction

Traumatic wounds are a common emergency in horses and are frequently coupled with sequelae that can be both career- and life-threatening [1,2]. Existing literature on equine wound healing consists predominantly of retrospective studies based in referral hospital populations, extrapolations from human medicine, and expert opinion [3].

Factors that have been described as affecting equine wound healing can be categorised into horse factors and wound factors. The main evidence in horse factors relates to differences between ponies and horses. Ponies have been reported as having faster rates of healing [4], higher levels of polymorphonuclear leucocytes, and more efficiently arranged fibroblasts [5] in experimental studies. They have also been reported to have a lower frequency of bone sequestra and wound dehiscence in a retrospective hospital study [6]. There is a larger body of evidence around wound factors. This includes the impact of wound location, as well as the involvement of other structures (including bone, tendon and synovial structures). The current evidence on wound location has described differences in healing between the trunk and limbs reported in experimental studies [7], and this is also frequently mentioned in expert opinion reviews [8,9]. A contributing factor may be the reduced distribution of musculature (including the panniculus muscle) on the equine distal limb. The involvement of critical anatomical structures affects both the duration of healing as well as the long-term outcome [2,10,11]. Again, most published studies are focused on referral hospital populations, rather than the wider population of horses. Wounds are frequently treated by veterinarians in primary care practice or managed entirely by horse owners themselves. This population of injuries is likely to comprise the majority of traumatic equine wounds and will be different in demographic and severity to those presented in referral hospitals. The aim of this study was to describe the causes, management and outcome of owner-reported traumatic equine wounds.

The study objectives were to: (1) collate data from horse owners on traumatic wounds occurring in the horse population from initial injury to final outcome and return to work; (2) describe the causes, presenting signs, owner- and veterinary-administered treatment, healing duration and outcome of traumatic equine wounds; (3) explore owner preparedness and decision-making regarding first aid and veterinary involvement for equine wounds.

## 2. Materials and Methods

The study was approved by the School of Veterinary Medicine and Science Clinical Ethical Review panel. All participants completed an informed consent form before being involved in the research (Supplementary Materials S1).

### 2.1. Study Design

A cohort study was conducted through a questionnaire distributed to owners/carers of equids which had suffered a traumatic wound. Data capture forms were designed to capture information relating to factors reported in the current literature to affect wound healing (including horse demographics, and the cause, diagnosis and treatment of the wound), and to monitor clinical progress from initial injury through to final healing and outcome (Table 1, Supplementary Materials S1 and S2).

### 2.2. Setting

This study captured owner-reported data on traumatic equine wounds between 1 October 2017–31 June 2019.

### 2.3. Participants

Eligibility criteria were horse owners/carers who were involved in the care of an equine that was currently suffering from, or had suffered within the preceding 12 months, any form of traumatic wound injury. Horse owners/carers had to be over 18 years of age to participate.

Participants were recruited using a snowball sampling method, carried out by both the University of Nottingham, UK, and the British Horse Society. The British Horse Society developed a dedicated website page, published promotional materials, and also advertised the study at 12 UK equestrian events. The British Horse Society and the University of Nottingham generated articles in 10 different equestrian-related magazines, various e-news articles and social media posts, and a press release for UK publication by the Veterinary Times. All equine veterinary practices registered with the Royal College of Veterinary Surgeons in 2017 were contacted via email and post to ask for their support in disseminating study awareness. Practices that responded and expressed interest were posted A5 flyers detailing the study to distribute.

### 2.4. Variables

Two data capture forms were developed and made available in both Google and Adobe electronic formats (https://docs.google.com/forms (accessed on 7 March 2019), and Adobe Acrobat Reader DC (2019)) (Table 1, Supplementary Materials S1 and S2). ‘Form 1’ collected data on the injured horse, initial wound assessment and any initial treatment received from the owner/carer or a vet (Table 1). ‘Form 2’ was completed when the wound had completely healed, except for any scarring, and focused on wound outcome (Table 1). If the wound had healed but the horse had not returned to the same capacity of work at the end of the study, then the owner was able to submit data on wound healing and when the horse was predicted to return to work. The survey questions were designed based on a review of current veterinary and medical literature on factors affecting wound healing in horses.

### 2.5. Data Sources/Measurement

Forms could be completed electronically, and data automatically submitted, or completed by hand and returned by email or post. All data included in this project were generated by horse owners, with no data submitted by veterinary surgeons. Data were collected using a structured form, with questions based on evidence reviews (Table 1, Supplementary Materials S1 and S2). Survey logic was used to direct participants to relevant sections for their horse’s injury.

### 2.6. Bias

Reporting bias was minimised by enabling owners to submit photographs of wounds in addition to written descriptions. These were reviewed by the primary researcher to verify wound location and type. Misunderstanding bias was minimised by including definitions, descriptions and/or diagrams for areas where knowledge may be required, including body condition scoring, location of wound, lameness assessment and types of bandages (Table 1). Involvement of tendon/ligament, bone or synovial structure was based on owner reporting of these complications and was not verified by veterinary clinical records; as a result, there may be reporting bias for these variables.

### 2.7. Statistical Methods

Data were transferred into an Excel spreadsheet for manual cleaning. Data were then transferred into SPSS version 25.0 and coded to allow for simple descriptive analysis of each question from the original data capture forms (IBM SPSS Statistics for Windows 2017, IBM Corp., version 25.0., Armonk, NY, USA). Questions were not compulsory, and therefore data are reported as frequency percentages (x/y), where y = number of completed responses for that question. Data were analysed as the median (interquartile range) for data that were not normally distributed, or the mode (range) for categorical data. Frequency percentages were reported as whole numbers; median and interquartile range were reported to one decimal point. Data responses for key variables were also grouped into categories and reported as the median (IQR), as follows:

Horse height: horses less than 14.3 hh were categorised as ponies, and horses more than 14.3 hh were categorised as horses.

Vaccination status: Vaccination status was categorised as appropriate when the owner selected the response ‘vaccine is up to date’. It was categorised as inappropriate when the owner selected responses either that their horse ‘has never been vaccinated’, ‘has been vaccinated but the vaccine is out of date’, or that they were ‘unsure of vaccination status’.

Wound locations were categorised as limb wounds (fore and hindlimb, proximal and distal locations) or non-limb wounds (all other locations).

Free text responses detailing first aid treatments, including topical treatments and bandaging, were reviewed and categorised by the primary researcher.

For reporting of outcome data, the number of horses in each category (e.g., horse vs. ponies, mares vs. geldings, limb or non-limb, tendon/ligament involved or not, synovial involvement or not, bone involvement or not, hospitalised or not) was determined and compared between the initial survey and the outcome survey to evaluate proportional follow up.

## 3. Results

### 3.1. Responses

**Figure 1:**
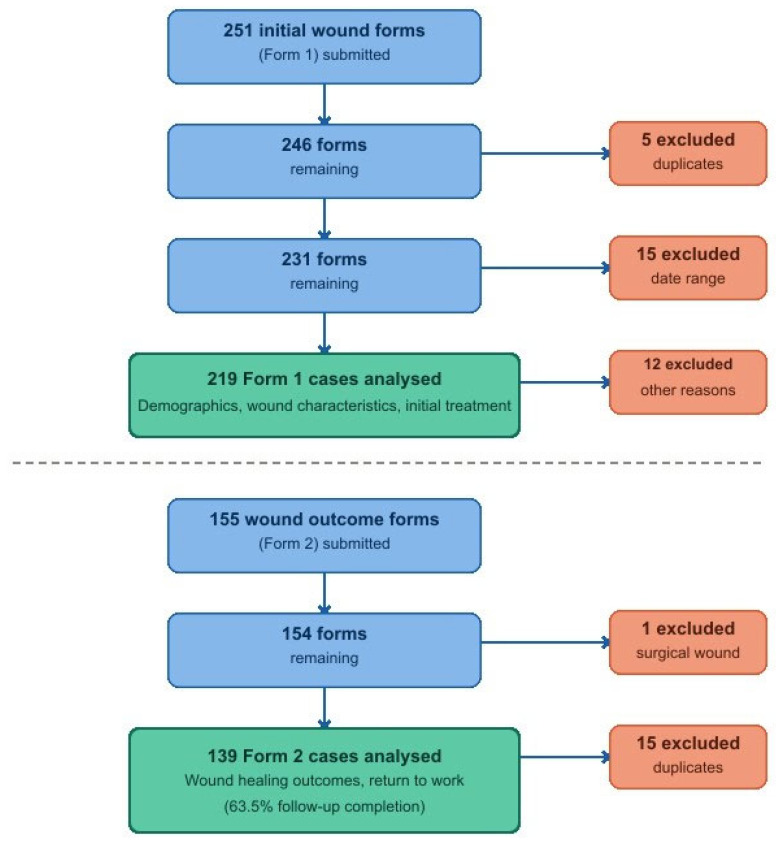
Flow chart of responses for an owner-reported cohort study of equine wounds in the UK.

A total of 251 responses were received to ‘Form 1’. A total of 32 responses were excluded from data analysis. Reasons for exclusion were duplicated forms (*n* = 16), outside of the accepted date range (*n* = 15), or surgical wounds (*n* = 1) (Figure 1). Twenty-five owners submitted photographs of their horse’s wounds. These were reviewed by the primary researcher against the wound location reported, and all were consistent with the data entered. Information on wound outcome (‘Form 2’) was received for 155 cases, where the wound had healed by the conclusion of the study. Five ‘Form 2’ responses were excluded due to duplication and 11 were excluded due to submission of retrospective cases outside of the accepted date range (Figure 1). A total of 219 wound case studies were therefore included, with outcome data analysed for 139 (64%). Cases were submitted online (Google Forms) (*n* = 190/219), using electronic forms (Adobe PDF) (*n* = 26/219), and on posted hard copies (*n* = 2/219).

### 3.2. Descriptive Data

#### 3.2.1. Horse Details

Most responses were received from the United Kingdom (95%, *n* = 201/211). Affected horses had a median age of 12.0 years (interquartile range, 7.0–30.0 years). The height of horses varied, with a mode height of 16–16.3 hands (range: 10–10.3 hands to more than 18 hands). There were 134/219 geldings (61%), and the most common breeds represented in this study were thoroughbreds (26%, *n* = 55/213), warmbloods (16%, *n* = 33/213) and Irish sport horses (15%, *n* = 32/213). The most frequently selected body condition score was ‘3’ (47%, *n* = 103/219). The most commonly indicated level of exercise that horses were involved in at the time of injury was a ‘light’ level of work (47%, *n* = 103/219), defined as ‘exercised three or four times weekly’.

There were 181/215 (84%) horses appropriately vaccinated against influenza and 92% (*n* = 196/214) appropriately vaccinated against tetanus. Sixteen percent (*n* = 36/219) of owners did not have access to equine transport facilities if their horse needed emergency referral/hospitalisation. Fifty-two percent of participants did not have their horse insured against veterinary fees (*n* = 114/219). For respondents who provided details regarding the level of insurance cover, the most commonly indicated policy value was £5000–£5999 (*n* = 41/78).

#### 3.2.2. Wound Details

When asked when the wound was first noticed, most owners (42%, *n* = 92/219) selected either afternoon (12 p.m.–6 p.m.), or morning (6 a.m.–12 p.m.) (42%, 91/219), with fewer selecting evening (6 p.m.–12 a.m.) (15%, *n* = 32/219) or nighttime (12 a.m.–6 a.m.) (2%, 4/219).

Twenty-seven cases had injuries affecting more than one anatomical location, with data reported on 251 wound locations. Most wounds occurred on a limb (67%, *n* = 168/251), with the majority of these on the hindlimb (38%, 97/251) (Table 2). Limb wounds were more frequently located in the distal limb than the proximal limb for both forelimb and hindlimb. The most frequently reported wound location was the distal hindlimb (31%, 79/251) (Table 2).

The most common cause of wounds was a wire/fence injury (38%, *n* = 84/219), followed by a kick injury (15%, *n* = 33/215), an unknown cause (11%, *n* = 25/251), and a stable injury (9%, *n* = 19/251) (Table 3). In the 24 h following injury, owners reported the degree of lameness as sound (35%, *n* = 54/153), not observed at walk but could be seen when turning (30%, *n* = 45/153), bearing less weight on the affected limb (26%, *n* = 40/153), and non-weight bearing (9%, *n* = 14/153). The proportion of wounds that participants thought definitely involved a ‘tendon or ligament’ was 17% (*n* = 28/169), for ‘bone’ was 16% (*n* = 27/164), and for ‘joint, tendon sheath or bursa’ was 16% (*n* = 26/166). Owners thought that the initial wound had no obvious contamination (46%, *n* = 101/219), dirt/soil contamination (42%, 93/219), obvious infection (9%, 19/219), contamination with another substance (7%, *n* = 16/219), or faecal contamination (3%, *n* = 6/219).

#### 3.2.3. Initial Wound Treatment

When asked about their initial wound treatment, 43% (*n* = 94/219) of participants administered first aid and called the vet within 24 h of injury, 32% (*n* = 71/219) called the vet immediately and did not administer first aid, 24% (*n* = 53/219) gave first aid only and did not call the vet, and two participants did not call the vet or administer first aid within the first 24 h.

There were 146 participants who reported administering first aid (defined as any form of wound cleaning, application of topical medications, or bandaging). Owners reported their hand preparation as washing with a disinfectant (e.g., chlorhexidine gluconate or povidone iodine) (27%, *n* = 38/143), washing with water only (26%, *n* = 37/143), not washing hands (25%, 36/143), washing with soap (15%, 22/143), wearing medical grade gloves (13%, *n* = 19/143), or applying an alcohol-based hand rub (2%, 3/143). Wound cleaning methods reported included washing the wound with tap or hosepipe water (25%, *n* = 37/146), washing with chlorhexidine (20%, 29/146), flushing with a hosepipe (14%, 21/146), washing with sterile saline (10%, 14/146), and flushing with a syringe (8%, 11/146). Of the 146 participants who administered first aid, 73 participants said they would not apply any topical treatments. The remainder completed a free text option detailing their treatment. The most common categories for topical medications, creams or powders applied to their horse wounds were ‘purple/blue spray’ (27%, *n* = 20/75), ‘Sudocrem’ (19%, *n* = 14/75), ‘silver ointment/Flamazine’ (15%, *n* = 11/75), ‘veterinary wound powder’ (13%, *n* = 10/75) and ‘manuka honey’ (9%, *n* = 7/75). Most participants chose not to apply a bandage (62%, *n* = 90/146). If they used a bandage, the most common primary contact layer (indicated in a free text box) was Melonin (59%, *n* = 23/39).

#### 3.2.4. Initial Veterinary Treatment

When asked about their confidence in deciding if veterinary treatment was required, 201 participants gave responses. Fifty-three percent (*n* = 106/201) of participants selected that they ‘had no doubt this wound needed veterinary attention’, 28% (*n* = 57/201) selected ‘I was confident this wound needed veterinary attention’, 10% (*n* = 19/201) selected that they were ‘equally unsure on whether to call the vet or not’, and 10% (*n* = 19/201) selected ‘I was not confident as to whether this wound required veterinary attention’.

For those wounds which were treated by a veterinary practitioner (*n* = 157), over half of the respondents stated that their veterinary practitioners did not apply any topical medications, creams or powders (55%, *n* = 86/157). The top five most commonly administered topical medications by veterinary practitioners (reported in free text comments and categorised) were ‘manuka honey’ (28%, *n* = 20/71), ‘silver ointment/Flamazine’ (24%, *n* = 17/71), ‘Dermagel’ (10%, *n* = 7/71), Intrasite (4%, *n* = 3/71) and ‘purple/blue spray’ (4%, *n* = 3/71). Nearly half of the cases treated by vets did not have a bandage applied (49%, *n* = 77/157). The most common primary contact layers were ‘Melonin’ (*n* = 34/80), and ‘Allevyn’ (*n* = 10/80).

Owners reported that the majority (98%, *n* = 154/157) of veterinary practitioners administered systemic medication. A total of 80% of cases (*n* = 124/157) received systemic antibiotics, 71% (*n* = 111/157) received systemic anti-inflammatories, and 62% (*n* = 98/157) received both. In 62% (*n* = 101/164) of cases, further procedures or investigations were not performed. There were 25 cases which were hospitalised, 24 of which received radiography, 15 of which had surgery, and of 12 which had ultrasonography.

#### 3.2.5. Outcome Data

Outcome data were provided for 139 horses. Wounds took a median of 60.0 days to heal (IQR 30.3–157.0). Healing was defined as when the wound had completely closed, except for any scarring. Three horses (2%) were euthanised as a direct result of their injury. A total of 13% of cases were never out of work throughout the period of injury (*n* = 17/135). A total of 47% (*n* = 63/135) of horses returned to the same level of work prior to complete healing, 24% (*n* = 33/135) were predicted to return to the same level of work, and 8% (*n* = 11/135) were not predicted to return to the same level of work at the conclusion of the study.

The time taken for horses to return to the same level of work was a median of 6.5 weeks (IQR 2.0–16.0). The median age was 12.3 years (IQR 8.0–17.0, *n* = 110) for horses returning to work, and was 20.0 years (IQR 13.0–25.0, *n* = 8) for horses not returning to work (Table 4). The median time for mares to return to work was 8.0 weeks (IQR 5.0–24.0, *n* = 33), and for geldings was 4.0 weeks (IQR 2.0–12.0, *n* = 62). The median time for ponies (less than 14.3 hh) to return to work was 8.0 weeks (IQR 1.0–12.0, *n* = 23) and for horses over 14.3 hh it was also 8.0 weeks (IQR 3.0–23.0, *n* = 62) (Table 4).

The median time to return to work was 6.0 weeks (IQR 2.0–12.0, *n* = 60) for limb wounds and 8.0 weeks (IQR 2.5–8.0, *n* = 36) for non-limb wounds (Table 4). The number of horses with wounds involving a tendon/ligament, synovial structure, or with severe lameness (Grade 5) returning outcome data at the conclusion of the study was low (12/28, 5/26, 6/14 respectively), and the numbers not returning outcome data was proportionately higher than wounds without these injuries/levels of lameness. The median and IQR data for this subset are therefore not reported.

The median cost of treatment was £900 (range: £0–7000). ‘Finances’ (10%, *n* = 14/139), ‘horse temperament’ (10%, *n* = 14/139) and ‘facilities’ (4%, *n* = 6/139) were the most common factors described to limit wound treatment.

## 4. Discussion

### 4.1. Key Results

This study used an owner-reported cohort approach to explore the outcomes of wound injuries in horses. Horse owners reported data on 219 equine wound injuries over a 10-month period; outcome data was available in 64% of cases. The demographics (sex, age, breed and height) of the horses in this study were similar to other studies reporting on the general horse population [12]. In terms of preparedness for managing wounds, 8% (*n* = 18/214) of horses were not vaccinated against tetanus and 16% of owners (*n* = 36/219) would not have access to equine transport should they require emergency treatment at a hospital. Fifty-two percent of horses in this study did not have insurance for veterinary fees (*n* = 114/219), and 10% (*n* = 14/139) of participants said their horse’s medical treatment was limited by cost. The most common cause of wounds was a wire/fence injury (38%, *n* = 84/219), and the most common site was the distal hindlimb (*n* = 79). This study captured data on a range of injuries and severities. A small proportion of wounds involved tendons/ligaments (*n* = 28), bone (*n* = 27) or synovial structures (*n* = 26). Most of the current literature describes equine wounds treated by veterinary practitioners [13,14,15], but this study gives a perspective on owner involvement and decision-making. The owner/carer administered first aid in most cases (67%, *n* = 147/219), and 75% (*n* = 165/219) of cases then received veterinary treatment. There is limited data on the duration of healing for wounds seen in the general horse population. In this study, the time taken for wounds to heal was a median of 60 (IQR 30.25–157) days, and the time taken to return to work was a median of 6.5 weeks, but these findings should be interpreted with caution due to the number of horses which did not return final outcome data, including several with more severe injuries.

### 4.2. Limitations

There are limited published data from primary care practices or on wounds that do not receive veterinary treatment. This study used an owner-reported approach to capture data around wound injury, treatment, and outcomes to capture this perspective. Nonetheless, this study format does have several limitations and potential biases; for example, there are possible reporting errors if the owner was not aware of or did not understand the complexity of the injury. Data on horse demographics, wound healing time and outcome may be more likely to be reliably reported by owners in comparison to veterinarian-reported data. However, retrieving data from veterinary clinical records and comparing this to the owner-reported data would have improved the reliability of our findings. The date of the injury was not recorded in this study, so any seasonal effects could not be investigated. This should be considered in future studies as this may affect pasture turn-out and workload.

The study duration was relatively short, and less severe injuries may be overrepresented in the final outcome data. The number of cases with wounds involving critical anatomical structures, more severe lameness, or requiring hospitalisation completing final outcome data was low and disproportionate to those completing the initial survey. A longer duration of study would be required to fully assess these cases.

The study design recruited owners through promotion utilising social media, vet practices and the British Horse Society, which introduces self-selection and sampling bias. The study does not represent the incidence or distribution of wounds within the wider population. Ideally, a cohort of owners would be recruited prior to injury, and horses monitored and case details completed if, and when, an injury occurred. However, as injuries are sporadic and unpredictable, this would require a very large cohort of horse owners to be recruited and remain engaged for a prolonged period of time to identify cases. This would provide an accurate representation of the incidence and type of wounds occurring within the general horse population.

Case recruitment and obtaining outcome data can prove difficult in cohort studies, with loss of participation/engagement commonly occurring. Despite extensive efforts to recruit cases, data were collected on a relatively small number of cases. In addition, outcome data were only available in 64% of cases despite a number of follow-up emails, and therefore attrition bias is also a study limitation.

### 4.3. Owner Preparedness for Managing Equine Wounds

This study identified several potential areas for improvement in terms of owner preparedness for equine wounds, including tetanus vaccination, emergency transport and financial planning. Only a small proportion of horses in this study were not vaccinated appropriately against tetanus (8%). However, tetanus has a high mortality rate and is easily preventable by vaccination [16]. The proportion of horses without appropriate vaccination cover in this study is comparable to a previous owner-reported study of the wider horse population [12]. The inappropriate tetanus vaccination cover in the current study is of particular concern as this relates to a group of horses which are at a higher risk of tetanus due to sustained wounds. In 16% of cases, the owners/carers did not have access to emergency transport for further treatment for their horse. The reasons for this were not explored, but could relate to finances and personal circumstances/opinions on referral treatment for their horse [17]. It is also likely that assistance or advice/potential contacts would be offered by friends/family or veterinary teams. Therefore, this finding reflects owner forward planning/preparedness rather than the ability to have the horse transported. Emergency planning should also include having a financial plan for the costs of veterinary treatment [18]. Over half of the cases were not insured for veterinary treatment, and approximately one in ten owners said that cost considerations limited their horse’s treatment. The total costs of treatment had a large range across the different cases, highlighting the variation in wound severity and treatments required. This, combined with the fact that wounds are unexpected emergency conditions, can make it challenging for horse owners, and again highlights the importance of having emergency plans and contingencies in place. A qualitative study of horse owners’ and veterinary practitioners’ experiences of critical decision-making in cases of colic reported that horse owners who had insurance were less anxious and concerned about discussing costs, and veterinary practitioners described them as being less concerned about finance and more aware of treatment costs [17]. Cost has also been reported as an important factor affecting referral treatment and euthanasia decisions for colic [19]. This highlights that having insurance cover can reduce the impact of finances/costs on owner worry and decision-making in emergency conditions, including colic and wounds. The behavioural framework COM-B model can be used to analyse human behaviour, evaluating Capability, Opportunity and Motivation [20]. In this study, both the lack of transport for a horse with an emergency condition, and not having insurance cover and/or finances to cover treatment costs both impact an owner’s capability to pursue referral treatment if required.

### 4.4. Owner Decision-Making and First Aid Treatment

The study identifies a number of areas where information or resources to support owners in decision-making and first aid could be beneficial. The majority (80%) of owners were confident in deciding whether veterinary treatment was required for their horse, but the one in five owners who were unsure or not confident are important to consider. Further studies to explore what types of wounds owners are not confident with, and what resources would support this, are important.

The most commonly selected response around wound contamination was ‘no obvious contamination’ (46%, 101/219). This is surprising considering the cause of most equine wounds and the environments in which they occur. It most likely reflects any gross contamination visible to owners, but may lead to false assumptions around actual contamination/microbial exposure. There are also areas for improvement/education around owner-administered first aid. There are multiple resources and guidance in human healthcare about the importance of handwashing with water and soap or with an alcohol-based handrub before healthcare [21]. In this study, 25% of owners did not wash their hands before giving first aid, and a further 26% washed them with water only. There may be a number of factors contributing to this, which again can be related to the COM-B model. These include not recognising the wound as contaminated (Motivation), and not having the facilities for appropriate handwashing at the location where the horse was injured (Opportunity). This study was conducted prior to the COVID-19 outbreak. It is likely that alcohol-based handrub is now both more available in different locations, and the importance of handwashing is more widely recognised, although behavioural changes regarding handwashing has been declining after 2020 [22].

The first aid applied to the wound warrants further investigation, with most owners reporting using tap water, a hosepipe, or chlorhexidine. Appropriate use of these will depend on the source of water, the volume and pressure of lavage solution, and the dilution of chlorhexidine [3]. Finally, there were only a small proportion of owners who reported on the topical treatment used, but again this highlights an area for further research and potential education/supporting information to inform choice. It was unknown how these topical treatments were applied, but a number of those listed can have potentially negative effects depending on how and when they are applied to open wounds, including purple/blue sprays, oil-based creams and wound powders (depending on the type of powder [23]) [24,25]. Further studies are required to understand which specific products are being used, and how they are being applied, along with guidance on appropriate products and use. The most common topical products applied by veterinary practitioners in this study were more likely to be honey or silver dressings, or hydrogels, with purple/blue spray used less frequently (*n* = 3). The differences between owner and veterinary practitioner choices highlight an opportunity for veterinary teams to work with horse owners to provide advice and guidance on preferred topical treatments for wounds.

### 4.5. Wound Outcomes

The majority of horses returned to work after their injury, but three were euthanised and 11 were predicted not to return to work. There were a wide range of values regarding wound heal duration and time taken to return to work. The average age of horses which did not return to work was high (median 20 years). Factors such as the presence of co-morbidities, or the owner’s choice to retire horses, are likely to have contributed to decision-making in older horses and warrant further investigation.

The median time to return to work was similar for horses and ponies, but ponies had a smaller interquartile range compared to horses (1–12 weeks compared to 3–23 weeks). This is consistent with previous studies reporting improved wound healing in ponies compared to horses. Studies have reported fewer complications, including faster second-intention wound healing in five ponies compared to five horses using a standardised experimental study design [4], and higher rates of successful primary closure and lower incidences of bone sequestra in a retrospective clinical case series involving 89 ponies and 422 horses [6].

In this study, wounds located on a limb had a greater interquartile range concerning time to return to athletic function compared to wounds not located on a limb (2–12 weeks compared to 2.5–8 weeks). Limb wounds present a higher risk of involving critical anatomical structures such as bone, tendons/ligaments, and synovial structures compared to trunk wounds. In the current study, involvement of these critical anatomical structures all increased the cost of treatment. The study population had a relatively small number of these cases compared to studies focusing on these specific injuries, and outcome data were not reported for a number of them. This means that this data should be interpreted with caution. The study does, however, add to the existing evidence demonstrating the complexity and cost of managing wounds involving critical anatomical structures.

## 5. Conclusions

This study documents owner-reported traumatic wounds in horses, including the cause and location of wounds, first aid administered, veterinary treatments, costs, and outcomes (including healing times and return to work). Participants self-selected to participate, and the number of horses involved limit the generalisability of these findings to the overall horse population. The study does, however, highlight several areas where further information and resources could support behavioural change in horse owners and potentially improve wound outcomes. These include the importance of planning ahead and preparing for wound emergencies, and the need for resources/guidance for horse owners regarding decision-making concerning veterinary intervention and administering first aid.

## Figures and Tables

**Table 1:** Structure and order of questions included in two data capture forms that were used to conduct an owner-reported cohort study of the causes, management and outcome of traumatic wounds in horses. Form 1 focused on the horse’s signalment and initial injury. Form 2 focused on wound outcome.

Section Title	Number of Questions	Information Collected
Form 1		
Introduction and consent	1	Introductory paragraph outlining the study, and the consent form. There was also a link to further information on the BHS website. Participants could not continue unless they consented to the conditions of participation.
Photograph submission	1	Option to upload photographs of the wound, including the date any photos were taken.
Details of horse	19	Horse details including signalment, country of residence, coat condition, temperament, BCS ^1^, level of work, and insurance/vaccination status.
Details of wound	9	Wound details including date/time of injury, mechanism of injury, location ^2^, contamination, tissues involved, and lameness ^3^.
Initial wound treatment	1	Survey logic question to direct owner/carer to relevant section depending on whether they called a vet or administered first aid themselves.
Owner first aid	14	Owner first aid details including date/time of first aid, hand preparation, haemostasis, wound cleaning, medications, and bandaging ^4^.
Veterinary treatment	18	Details of any veterinary treatment administered, including date/time of treatment, sedation, owner confidence in seeking veterinary advice/treatment, haemostasis, cleaning, medications, bandaging, additional procedures, and cost limitations.
Euthanasia	2	Data on whether euthanasia was carried out as a result of the wound.
Form 2		
Introduction and consent	1	Information about this second form, and a check that participants had completed Form 1 and therefore given consent.
Photograph submission	1	Same as for Form 1.
Details of horse	3	Signalment of horse. This was included again so that different form submissions could be matched to each case.
Wound outcome		Outcome data including how long the wound took to heal, whether euthanasia was required, whether the owner was satisfied with the rate of healing, any return to work, lameness, cosmetic outcome, complications, limitations, what resources the owner would like about wound management, and how the owner heard about the study.
Prize Draw	1	Yes/No if they wished to be entered into this.

^1^ Participants were asked to indicate the Body Condition Score (BCS) of their horse using an infographic scale developed by the National Equine Welfare Council, who gave permission for the EWP to use the same scale in this study. The scale ranged from ‘0—very poor’ to ‘5—very fat’, with a corresponding diagram for each point on the scale. In this study, the number and descriptors were omitted, leaving just the diagram of each horse so as not to cause participants to be biassed in their response by selecting a BCS they believe their horse should be as oppose to what it actually was. ^2^ To help owners determine the location of their horse’s wound, a diagram was supplied which divided the anatomy of the horse into nine sections. ^3^ A lameness scale was designed for owners to safely assess, at the time of injury, whether their horse was exhibiting any lameness should the wound have occurred on a limb (*n* = 153). The scale was different to a traditional veterinary lameness scale, excluding trotting, to prevent owners exercising horses on a potentially compromised limb. ^4^ A heavy bandage was described as applying pressure to the wound with several layers of different material whilst a light bandage was described as simply covering the wound with a single material layer.

**Table 2:** Anatomical location of wounds from an owner-reported cohort study of the causes, management and outcome of traumatic wounds in 219 horses. The study questionnaire supplied participants with a diagram outlining nine different regions to help participants identify which region the wound affected. Twenty-seven cases had more than one wound location; therefore, the total number of wound locations reported was 251.

Anatomic Location	Number of Responses (%)
Head	27 (11%)
Neck	4 (2%)
Shoulder	26 (10%)
Trunk	13 (5%)
Hind	13 (5%)
Forelimb (above carpus/knee)	28 (11%)
Forelimb (below carpus/knee)	43 (17%)
Hindlimb (above tarsus/hock)	18 (7%)
Hindlimb (below tarsus/hock)	79 (31%)

**Table 3:** Causes of wounds from an owner-reported cohort study of the causes, management and outcome of traumatic wounds in 219 horses.

Cause of Wound	Number of Responses (%)
Wire/fence injury	84 (38%)
Kick	33 (15%)
Unknown	25 (11%)
Stable injury	19 (9%)
Fall	10 (5%)
Gate catch injury	8 (4%)
Puncture/penetration	8 (4%)
Overreach injury	8 (4%)
Other	7 (3%)
Horsebox injury	5 (2%)
Self-irritation	4 (2%)
Bite	4 (2%)
Dog bite	2 (1%)
Road traffic accident	2 (1%)

**Table 4:** Wound outcomes (from an owner-reported study of the causes, management and outcomes of traumatic wounds in 219 horses).

Outcome Measure	Variable	Additional Information
Number of days taken for the wound to heal	Received veterinary treatment	The median number of days it took for wounds that had received veterinary treatment to heal was 96.5 days (IQR 39.5–176.6, *n* = 81), and 44.0 days (IQR 20.8–85.5, *n* = 31) for those that did not receive treatment.
Return to previous work	Horse age	The median age for horses returning to work was 12.3 years (IQR 8.0–17.0, *n* = 110) and for horses not returning to work was 20.0 years (IQR 13.0–25.0, *n* = 8).
Number of weeks taken for a horse to return to previous work	Horse sex (gelding vs. mare/filly)	The median time it took for mares to return to work was 8.0 weeks (IQR 5.0–24.0, *n* = 33) and for geldings it was 4.0 weeks (IQR 2.0–12.0, *n* = 62).
	Horse height (horse vs. pony)	The median time it took for ponies (under 14.3 hh) to return to work was 8.0 weeks (IQR 1.0–12.0, *n* = 23), and for horses (over 14.3 hh) it was also 8.0 weeks (IQR 3.0–23.0, *n* = 72).
	Limb wound (limb vs. non-limb location)	The median time it took for horses with limb wounds to return to work was 6.0 weeks (IQR 2.0–12.0, *n* = 60), and for non-limb wounds it was 8.0 weeks (IQR 2.5–8.0, *n* = 36).
	Received veterinary treatment	The median number of weeks it took for horses to return to work that had received veterinary treatment was 8.0 weeks (IQR 3.0–20.0, *n* = 81), and for those that did not receive veterinary treatment it was 2.0 weeks (IQR 0.5–8.0 weeks, *n* = 31).
Total cost of treatment	Access to emergency equine transport	The median cost of treatment for horses who did have access to emergency equine transport was £1015 (IQR 295.0–2500.0, *n* = 88) and for those who did not was £300 (IQR 135.0–504.5, *n* = 21).
	Insured	The median cost of treatment for insured horses was £575 (IQR 233.0–2312.5, *n* = 52) and for uninsured horses it was £900 (IQR 265.0–2000.0, *n* = 57).
	* Tendon/ligament involvement	The median total cost of treatment for wounds involving a tendon or ligament was £2750 (IQR 1320.0–4437.0, *n* = 19), and for wounds not involving a tendon or ligament it was £509 (IQR 187.5–1900.0, *n* = 56).
	* Bone involvement	The median cost of treatment for wounds involving bone was £2500 (IQR 158.0–5000.0, *n* = 16), and for those not involving bone it was £900 (IQR 225.0–3250.0, *n* = 55).
	* Synovial involvement	The median cost for wounds involving a synovial structure was £1200 (IQR 175.0–3750.0, *n* = 18), and for those not involving a synovial structure it was £1200 (IQR 250.0–3000.0, *n* = 58).
	Received veterinary treatment	The median cost of treatment for wounds that received veterinary treatment was £840 (IQR 250.0–2050.0, *n* = 83), and £500 (IQR 262.0–2125.0, *n* = 26) for those that did not.
	Hospitalisation	The median cost of treatment for hospitalised horses was £400 (IQR 146.3–1375.0, *n* = 16), and for non-hospitalised horses it was £900 (IQR 250.0–250.0, *n* = 93).

* Indicates where data analysis was only performed on wounds located on a limb. Data is reported as the median (IQR) and the number of horses in each category as *n*.

## Data Availability

The data presented in this study are available on request from the corresponding author (participant informed consent was given for data use for research purposes only; therefore, data are not openly available, but are available from the authors upon reasonable request which complies with this consent).

## Supplementary Materials

The following supporting information can be downloaded at: https://www.mdpi.com/article/10.3390/ani16101474/s1, Supplementary Material S1. Form 1. Details of horse, initial wound assessment and any initial first aid/veterinary treatment. Supplementary Material S2. Form 2. Wound outcome.
