# Supplementary material for: Owner-Reported Cohort Study of Causes, Management and Outcome of Traumatic Wounds in 219 Horses
Source: Animals (Basel). 2026 May 11;16(10):1474. doi: 10.3390/ani16101474 (PMC13203199; doi:10.3390/ani16101474)
Supplement: Supplementary file 1 [file animals-16-01474-s001.zip › Supplementary Materials S1. Form 1.pdf]

Form 1: Details of horse*, initial wound assessment and any initial first aid/veterinary treatment

Please complete this form after you first noticed the wound on your horse, following any initial first aid given by yourself (the owner) or by a vet. Please only include any veterinary treatment given within 24 hours of noticing the wound. For any veterinary treatment given longer than 24 hours after injury, please input this information into form 2b. You will be asked to provide your email address, this will only be used to send reminders to complete follow up forms on the wounds healing progress.

*In this form the term 'horse' covers all horses, donkeys and hybrids

* Indicates required question

Consent

Please read the document 'Participation information' which can be found at www.bhs.org.uk/wounds. After considering the study information provided please read each of the following statements:

- I am over the age of 18 years old
- I have had the opportunity to read the provided information describing the above study
- I have had the opportunity to consider this information and ask any questions I may have had about this study and my involvement
- I understand that all information collected by The University of Nottingham will be kept strictly confidential and anonymised so that it will not be possible to identify any participants of this study
- I understand that I can refuse to take part if I wish, without providing a reasons, and this refusal will not affect my ability to take part in future studies
- I understand that even if I have consented to participate, I can withdraw from this study at any time without providing a reason
- I know that I can contact the researchers involved in this study for further information
- I understand that anonymised data collected during this study will be used as part of an MRes qualification, published within future scientific publications and be used to develop educational resources. This includes pictures submitted in this study.
- I give consent that any photos I submit as part of this study can be used in future scientific publications and advertising relating to this project and I understand that any photos used will remain anonymous
- I understand that by giving consent in this form ('Form 1'), I have therefore given the same consent for all data submitted in subsequent follow up forms associated with this study (Forms 2a, 2b & 3)

1. Do you agree with the above statements? *

Mark only one oval.

- [ ] I Agree

Photo submission

If you have taken any photos of your horse’s wound and are happy to do submit them as part of this study we would be very grateful as submission of photos are incredibly valuable data. Using an evidence based wound scoring system developed by a Nottingham Vet School undergraduate we hope to model different aspects of equine wound healing and predict how long different types of wounds take to heal. Please only restrain and take photographs of your horse if it is safe to do so and your horse's temperament allows. Do not place yourself, your horse or anyone else helping to handle the horse in any unnecessary danger. If it is impractical/unsafe to take photographs please remember to still submit the online forms.

If you do not have any photos of the wound or would not like to submit them, please do not worry! It would be great if you could continue to complete the rest of the form either way.

2. Photographs of the wound from a distance

   Files submitted:

3. Close up photographs of the wound

   Files submitted:

4. Date any photographs were taken

   ____

   Example: January 7, 2019

Details of horse

This section is all about collecting information about your horse itself, including its name, age and gender etc...

5. What is the name of your horse?

   This information will be kept anonymous and is only required so researchers can match up responses from different surveys

   ____

6. What is the age of your horse?

7. What gender is your horse?

Mark only one oval.

- Gelding
- Stallion
- Mare

8. What breed is your horse?

9. What height is your horse?

Mark only one oval.

- Less than 9 hands
- 9 - 9.3 hands
- 10 - 10.3 hands
- 11 - 11.3 hands
- 12 - 12.3 hands
- 13 - 13.3 hands
- 14 - 14.3 hands
- 15 - 15.3 hands
- 16 - 16.3 hands
- 17 - 17.3 hands
- More than 18 hands

10. What country does your horse live in?

11. Which picture do you believe best represents your horse's body condition at the time of injury?

These pictures were taken from resources developed by the National Equine Welfare Council's Compendium for horses and the Donkey Sanctuary for donkeys. They were designed to help owners body condition score their horses/donkeys. Further details can be found by following the link below:
http://www.newc.co.uk/advice/welfare/compedium/

Check all that apply.

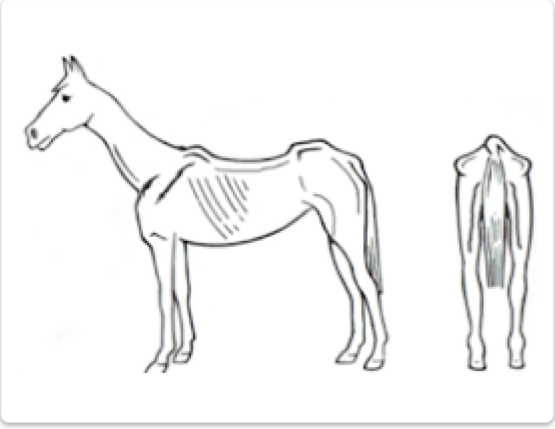

☐ Horse option 1

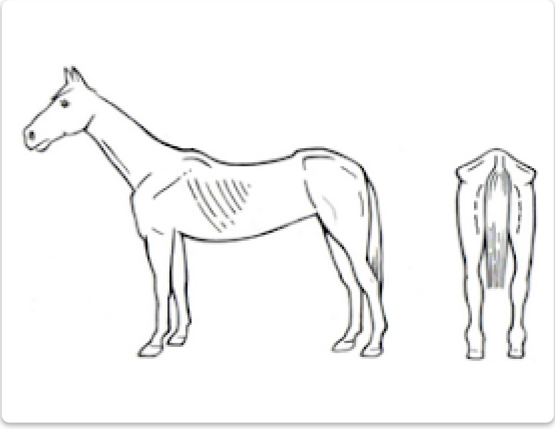

☐ Horse option 2

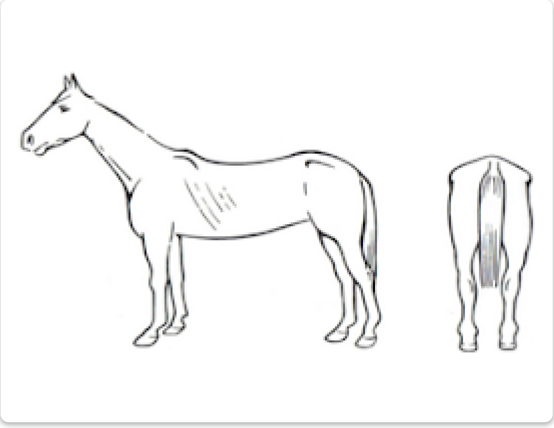

☐ Horse option 3

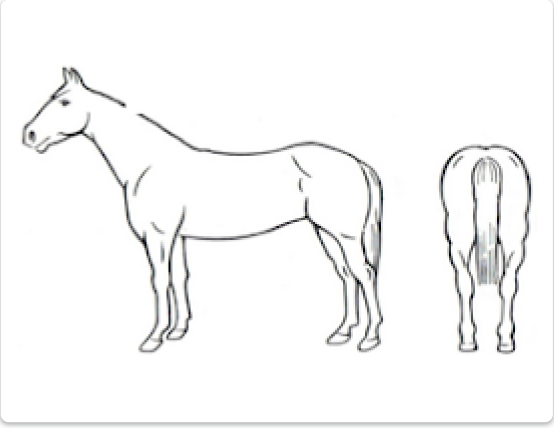

☐ Horse option 4

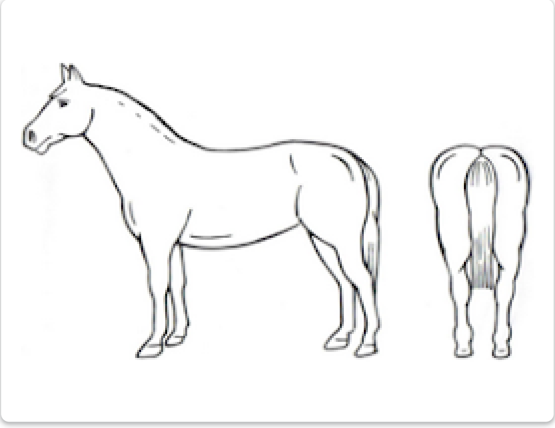

☐ Horse option 5

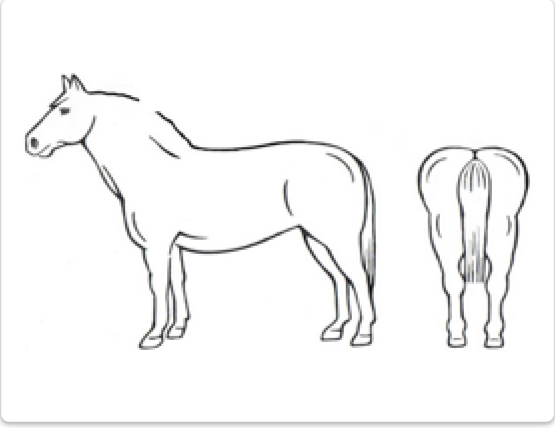

☐ Horse option 6

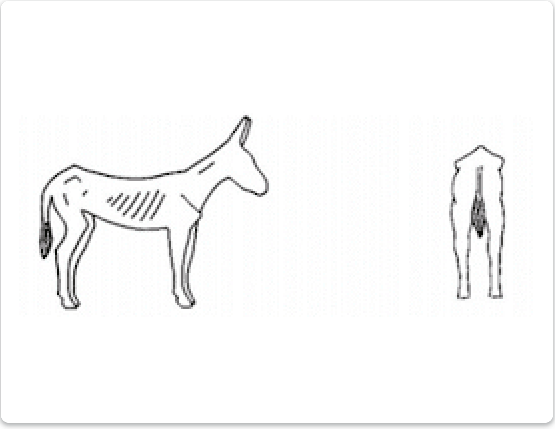

☐ Donkey option 1

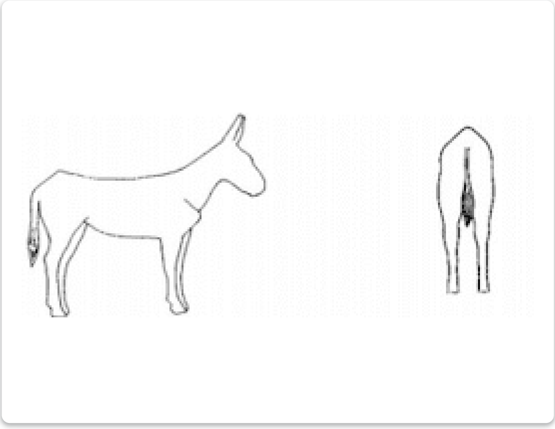

☐ Donkey option 2

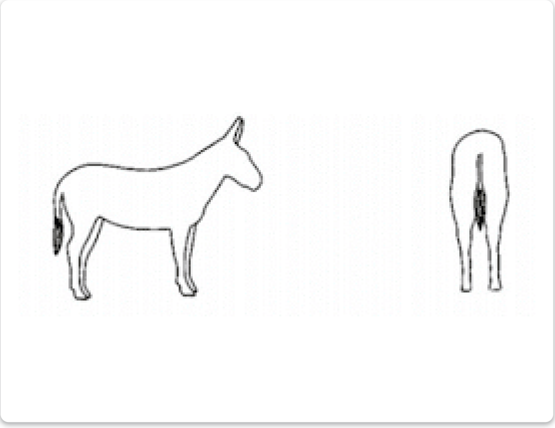

☐ Donkey option 3

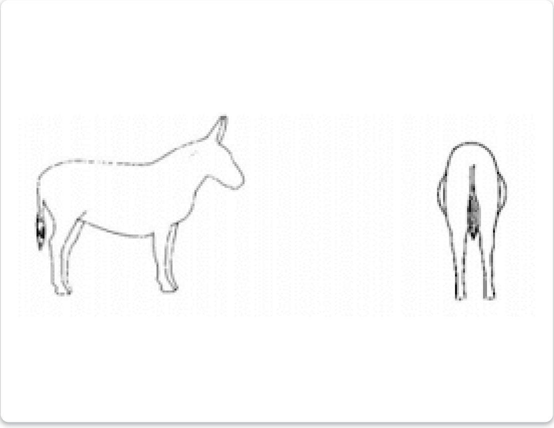

☐ Donkey option 4

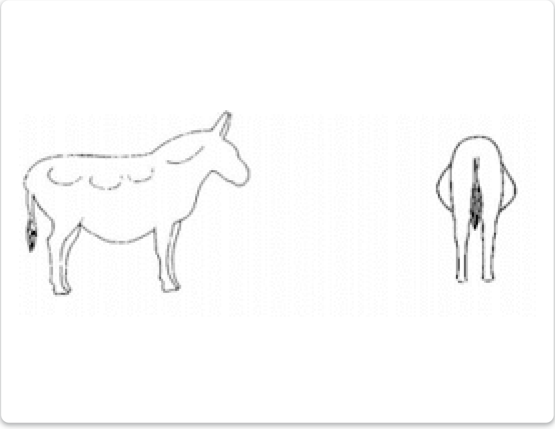

☐ Donkey option 5

12. Was the area around your horse's wound prior to injury clipped or unclipped?

Mark only one oval.

- Clipped
- Unclipped
- Other: ____

13. How would you describe your horse's coat cleanliness at the time of injury?

Mark only one oval.

- Very clean
- Moderately clean
- Fairly dirty
- Filthy

14. Thinking about your horse, which of the following best describes your horse's temperament on a daily basis?

Mark only one oval.

- Docile (Horse is very easy and safe to handle and restrain)
- Average (Horse is safe enough to be handled by most people but will sensibly spook at certain stimuli, e.g. a loud bang)
- Anxious (Horse is energetic and more likely to spook, requires an experienced handler for restraint)
- Highly Strung (Horse cannot be safely restrained for simple procedures, e.g. being shod, without prior sedation)

15. What is your horse's current vaccination status?

Check all that apply.

	Never been vaccinated for	Has been vaccinated for but vaccine is out of date	Vaccine is up to date	Unsure
**Equine influenza**	☐	☐	☐	☐
**Tetanus**	☐	☐	☐	☐

16. What is the main activity carried out by your horse?

Check all that apply.

- ☐ Dressage
- ☐ Show-jumping
- ☐ Eventing
- ☐ Showing
- ☐ Horseball
- ☐ Racing / point-to-point
- ☐ Polo
- ☐ Endurance
- ☐ Breaking in / training
- ☐ Hacking
- ☐ Schooling
- ☐ Western
- ☐ Vaulting
- ☐ Companion - grooming, handling etc...
- ☐ Riding club
- ☐ Mounted games
- ☐ Retired
- ☐ Trec
- ☐ Breeding
- ☐ Jumpcross / polocrosse
- ☐ All rounder
- ☐ Working equitation
- ☐ Gaited competition
- ☐ Other: ____________________

17. What level is your horse working at?

Describe what level of work/competition your horse was at immediately prior to wound injury

Mark only one oval.

- None
- Light (Exercised three or four times weekly)
- Moderate (Participate in a near daily structured training programme)
- Heavy (Train/compete at the peak of their physical abilities, e.g. thoroughbred racehorses)

18. Has your horse ever been diagnosed with Cushings disease (PPID)?

Diagnosis is confirmed by a vet taking a blood test. Further information can be found on the following link: http://www.bhs.org.uk/welfare-and-care/horse-health-and-sickness/cushings-disease

Mark only one oval.

- Yes
- No
- Unsure

19. Is your horse currently on any medications?

Mark only one oval.

- Yes
- No
- Unsure

20. If your horse is on any medication, please give further details. Particularly if your horse is on corticosteroids (steroids).

If your horse is currently on any medication please include the medication name, how long the horse has been taking it, how often it takes the medication and for what reason (e.g. 30 prednisolone tablets daily for the last four months to help manage breathing problems). If your horse is not currently on any medication please leave this question blank

21. If your horse required emergency treatment at a hospital, would you have access to emergency equine transport?

Mark only one oval.

- Yes
- No

22. Is your horse insured for vet fees?

Mark only one oval.

- Yes
- No

23. If your horse is insured for vet fees, how much cover do you have on your current policy?

If your horse is not insured, or you would prefer not to answer this question please skip to the next section

Details of the wound

24. Date that the horse injured itself

If you can't remember the exact date then please put an approximate date

____

Example: January 7, 2019

25. When did you first notice the injury?

Mark only one oval.

- ◯ 12am - 6am (Night)
- ◯ 6am - 12pm (Morning)
- ◯ 12pm - 6pm (Afternoon)
- ◯ 6pm - 12am (Evening)

Please use this image to help answer the next question

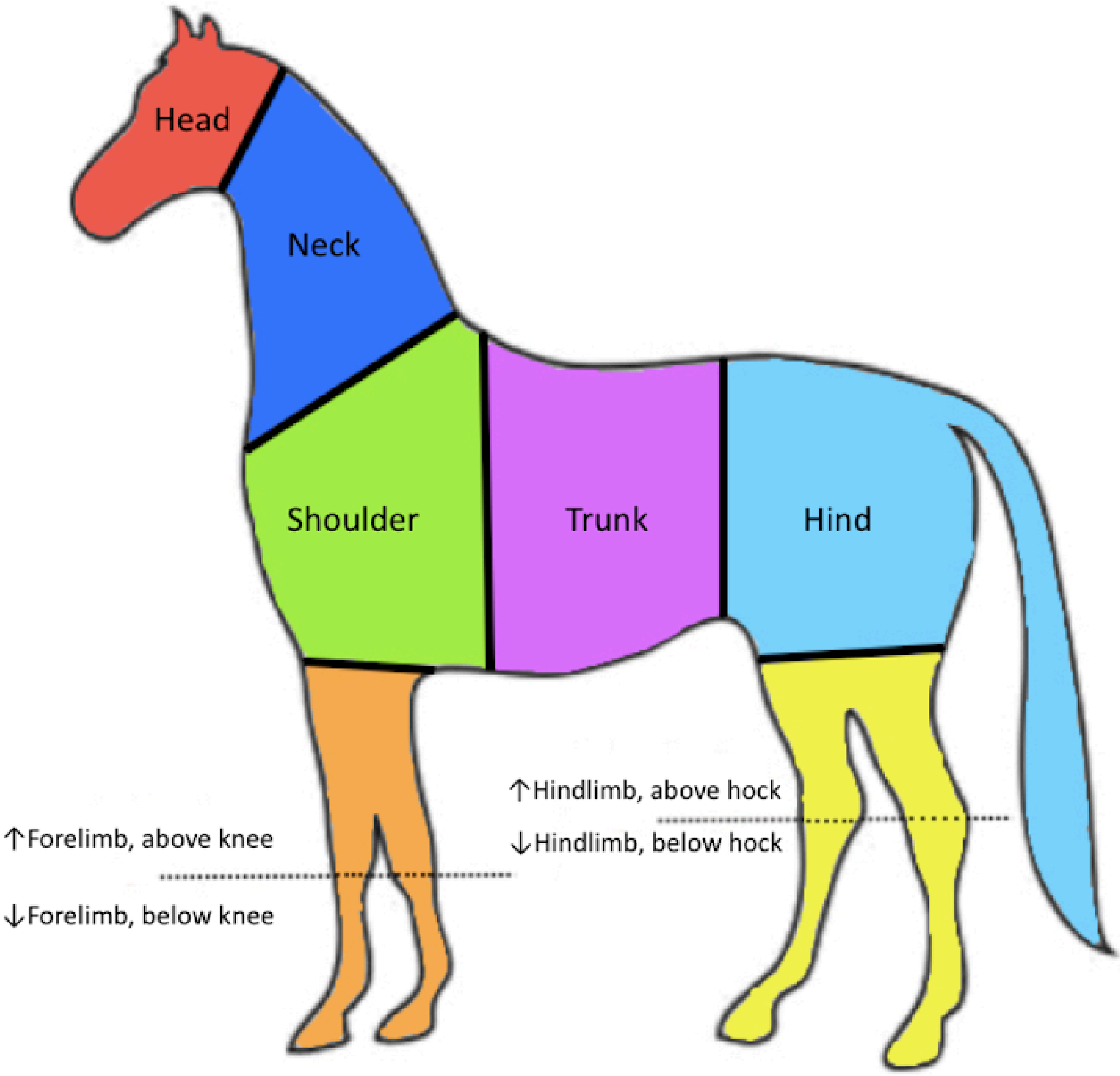

26. Where is the wound located on the horse?

Check all that apply.

- [ ] Head
- [ ] Neck
- [ ] Shoulder
- [ ] Trunk
- [ ] Hind
- [ ] Forelimb (above knee)
- [ ] Forelimb (below knee)
- [ ] Hindlimb (above hock)
- [ ] Hindlimb (below hock)

27. Is there any more information you would like to include on the wound location?

For example, on the front aspect of the cannon bone

28. For each of the following, indicate any tissues you believe to be involved in the wound

Check all that apply.

	Yes	No	Unsure
**Skin**	☐	☐	☐
**Muscle**	☐	☐	☐
**Tendon/ligament**	☐	☐	☐
**Bone**	☐	☐	☐
**Joint/tendon sheath**	☐	☐	☐
**Thoracic/abdominal cavity**	☐	☐	☐

29. What was the cause of the wound?

Check all that apply.

- [ ] Unknown
- [ ] Kick
- [ ] Con
- [ ] Field injury, e.g. wire/fence injury
- [ ] Stable injury
- [ ] Fall
- [ ] Road traffic accident
- [ ] Self irritation (E.g. from mud fever/sweet itch)
- [ ] Other: ______________________

30. Please provide any further details on the mechanism of injury

E.g. Horse tried to escape from its paddock and in the process lacerated its front right lower limb on barbed wire

____

____

____

____

____

31. Please state the wound's contamination level

Check all that apply.

- [ ] No obvious contamination
- [ ] Faeces contamination
- [ ] Dirt/soil contamination
- [ ] Contamination with another substance
- [ ] Obvious infection
- [ ] Other: ______________________

32. Grade any lameness caused as a result of the wound

Please only detail any lameness seen within the first 24 hours following initial injury

Mark only one oval.

- No lameness resulted because of the wound / horse is sound
- Cannot see lameness at walk and it is sometimes but not always there under certain circumstances (e.g. when turning)
- Lameness is difficult to observe at walk but is always there under certain circumstances (e.g. uncomfortable when turning)
- Horse is bearing less weight on the affected limb at walk/when resting
- Horse cannot bear weight on the affected limb

Initial wound treatment

33. Please explain if you gave any first aid immediately after finding the wound

Mark only one oval.

- Yes, I did give some initial first aid treatment and called the vet
- Yes, I did give some initial first aid treatment but did not call the vet
- No, I did not give any first aid as I called for the vet as soon as noticing the wound *Skip to question 48*
- No, I did not give any first aid and did not immediately call the vet after noticing the wound *Skip to question 67*

Initial first aid given by the owner

34. What date did you initially give first aid to the wound?

Example: January 7, 2019

35. What time did you initially give first aid to the wound?

____

Example: 8:30 AM

36. Did you prepare your hands before treating the wound?

Check all that apply.

- [ ] Did not wash hands
- [ ] Washed hands with water only
- [ ] Washed hands with soap
- [ ] Washed hands with a disinfectant (E.g. hibiscrub, iodine)
- [ ] Applied an alcohol based hand rub
- [ ] Wore medical grade gloves to treat the wound
- [ ] Other: _______________________________

37. Did you have to stop any bleeding?

Check all that apply.

- [ ] No
- [ ] Yes, by applying pressure with just my hands
- [ ] Yes, by applying pressure with cotton wool
- [ ] Yes, by applying pressure using a bandage
- [ ] Yes, by applying pressure with something else (e.g. towel)
- [ ] Other: _______________________________

38. Did you attempt to clean the wound? Please tick all of the following that apply

Check all that apply.

- [ ] No
- [ ] Yes, with tap/hosepipe water
- [ ] Yes, with cooled boiled water
- [ ] Yes, with hibiscrub (chlorhexidine)
- [ ] Yes, with iodine
- [ ] Yes, with sterile saline
- [ ] Yes, with another solution
- [ ] Flushed the wound using a hosepipe
- [ ] Flushed the wound using a syringe
- [ ] Scrubbed with cotton wool
- [ ] Scrubbed with swabs
- [ ] Scrubbed with another material
- [ ] Other: ____________________

39. If you used a solution other than water to clean the wound, how did you prepare/dilute the solution?

____

____

____

____

____

40. Please indicate any creams or powders you applied to the wound

If you applied a cream/powder that is not listed, please specify its name in the 'other' section

Check all that apply.

- [ ] Did not apply any creams/powders to the wound
- [ ] Purple/blue spray
- [ ] Manuka honey
- [ ] Derma gel
- [ ] Veterinary wound powder
- [ ] Aloe vera
- [ ] Silver ointment
- [ ] Sudocreme
- [ ] Filta bac
- [ ] SCP spray
- [ ] Other: ____________________

41. Did you administer any medication to the horse upon noticing the wound?

Mark only one oval.

- ( ) Yes
- ( ) No

42. If you administered any medication to your horse upon noticing the wound, please give further details

For example, 1/2 a sachet of oral phenylbutazone ('bute')? If you did not, please leave this section blank.

____

43. Did you apply any form of bandage to the wound?

Mark only one oval.

- No
- Yes, a light bandage to cover the wound (e.g. used adhesive tape to secure a primary contact layer to the wound)
- Yes, a heavier bandage to apply pressure to the wound (e.g. used vetrap)
- Other:

44. If you applied a bandage to the wound, what was the name of the material in direct contact with the wound surface?

E.g. primapore/melolin (if unsure the name can be found on its packaging)

45. Give details of any other actions you took to initially manage the wound?

46. Are you going to rest your horse?

If yes give details e.g. out of work for at least two weeks, box rest for one week, turned out into a restricted paddock for a week. If you are not resting your horse, please state this

47. Do you have a plan on how you are going to manage this wound?

E.g. How often you plan to change the dressings/wash the wound

Details of any initial veterinary treatment

This section is to collect information on any veterinary treatment the wound received within the first 24 hours after the owner noticed the wound. If the horse received veterinary treatment after 24 hours of you noticing the wound, please complete form 2b. If you did not call the vet please skip to the next section.

48. How confident were you that this wound needed veterinary attention?

Mark only one oval.

- I was not confident on whether this would need veterinary attention
- Equally unsure on whether to call the vet or not to call the vet
- I was confident this wound needed veterinary attention
- I had no doubts that this wound needed veterinary attention

49. Time of initial veterinary treatment

Mark only one oval.

- 12am - 6am (Night)
- 6am - 12pm (Morning)
- 12pm - 6pm (Afternoon)
- 6pm - 12am (Evening)

50. Did the vet have to sedate your horse to carry out any treatment?

Mark only one oval.

- ◯ Yes
- ◯ No
- ◯ Unsure

51. Did the vet have to stop any bleeding?

Check all that apply.

- ☐ No
- ☐ Yes, by applying pressure with their hands
- ☐ Yes, by applying pressure with cotton wool
- ☐ Yes, by applying pressure using a bandage
- ☐ Yes, by surgically stopping bleeding (e.g. using suture material to tie off vessels, applying clamps to bleeding vessels)
- ☐ Unsure
- ☐ Other: ______________________

52. Did the vet clip the hair surrounding the wound?

Mark only one oval.

- ◯ Yes
- ◯ No
- ◯ Unsure

53. If the vet cleaned the wound, please tick all the following which apply:

Check all that apply.

- [ ] Vet did not clean the wound
- [ ] Washed with tap/hosepipe water
- [ ] Washed with hibiscrub (chlorhexidine)
- [ ] Washed with iodine
- [ ] Washed with sterile saline
- [ ] Washed with another solution
- [ ] Flushed using a hosepipe
- [ ] Flushed using a syringe
- [ ] Scrubbed with cotton wool
- [ ] Scrubbed with swabs
- [ ] Scrubbed with another material
- [ ] Unsure
- [ ] Other: ____________________

54. Did the vet attempt to close the wound?

Check all that apply.

- [ ] Vet did not close the wound
- [ ] Vet sutured/stitched the entire wound closed
- [ ] Vet sutured/stitched part of the wound closed and left part open to drain
- [ ] Vet stapled the entire wound closed
- [ ] Vet stapled part of the wound closed and left part open to drain
- [ ] Vet both sutured/stitched and stapled the wound
- [ ] Unsure
- [ ] Other: ____________________

55. Please indicate any creams or powders the vet applied to the wound

If a cream/powder was applied that is not listed, please specify its name in the 'other' section

Check all that apply.

- [ ] Vet did not apply any creams/powders to the wound
- [ ] Purple spray
- [ ] Manuka honey
- [ ] Derma gel
- [ ] Veterinary wound powder
- [ ] Aloe vera
- [ ] Silver ointment
- [ ] Sudocreme
- [ ] Filta bac
- [ ] SCP spray
- [ ] Unsure
- [ ] Other: ____________

56. Did the vet apply any form of bandage to the wound?

If the vet did not apply a bandage please skip the next three questions

Mark only one oval.

- ( ) No
- ( ) Yes, a light bandage to cover the wound (e.g. used adhesive tape to secure a primary contact layer to the wound)
- ( ) Yes, a heavier bandage to apply pressure to the wound (e.g. used vetrap)
- ( ) Unsure
- ( ) Other: ____

57. If the vet applied a bandage to the wound, what was the name of the material in direct contact with the wound?

E.g. primapore/melolin (if unsure the name can be found on its packaging)

____

58. Date of next bandage change/removal

____

Example: January 7, 2019

59. Who will be doing the next bandage change/removal?

Mark only one oval.

- Vet
- Owner
- Other: ____

60. Did the vet prescribe any medications to be given by the owner?

Mark only one oval.

- Yes
- No
- Unsure

61. Please give the names of any prescribed medications given by the vet

E.g. Trimediazine (trimethoprim sulfadiazine) sachets, equipalazone ('bute') sachets, if you can please state the dose and duration (e.g. 1/2 sachet twice daily for four days)

____
____
____
____
____

62. Indicate any further procedures that were carried out

Check all that apply.

- [ ] No other procedures were carried out
- [ ] Radiography
- [ ] Ultrasonography
- [ ] Hospitalisation
- [ ] Surgery
- [ ] Other: ______________________

63. Did the vet give you any instructions on how to rest your horse?

If yes give details e.g. out of work for at least two weeks, box rest for one week, turned out into a restricted paddock for a further week

____
____
____
____
____

64. Please give details of any follow up treatment

E.g. How often the dressings will be changed, how often washing the wound, predicted follow up visits etc...

____
____
____
____
____

65. Were your treatment options limited by cost?

Mark only one oval.

- Yes
- No
- Prefer not to say

66. If you are happy to give the following information, what was the cost of veterinary treatment on this occasion?

____

Euthanasia

67. Was euthanasia required as a result of the wound?

Mark only one oval.

- Yes
- No
- Euthanasia was discussed/considered

68. If euthanasia was required on this occasion and you are happy to provide further details, please explain why euthanasia was opted for

For example, due to the cost of treatment/severity of wound/age of horse etc...

____
____
____
____
____

This content is neither created nor endorsed by Google.
