# Supplementary material for: Owner-Reported Cohort Study of Causes, Management and Outcome of Traumatic Wounds in 219 Horses
Source: Animals (Basel). 2026 May 11;16(10):1474. doi: 10.3390/ani16101474 (PMC13203199; doi:10.3390/ani16101474)
Supplement: Supplementary file 1 [file animals-16-01474-s001.zip › Supplementary Materials S2. Form 2.pdf]

Form 2: Wound outcome

Please complete this form when you believe the wound to have completely healed (apart from any remaining scarring).

* Indicates required question

1. Have you previously completed 'Form 1: Details of horse*, initial wound assessment and any initial first aid/veterinary treatment' *

   It is important for this study that you initially complete form 1 before proceeding with subsequent forms. Form 1 contains essential information on horse owner participation and consent. Further information can be found out at www.bhs.org.uk/wounds

   Mark only one oval.

   ◯ Yes

Photo submission

If you have taken any photos of your horse's wound and are happy to do submit them as part of this study we would be very grateful as submission of photos are incredibly valuable data. Using an evidence based wound scoring system developed by a Nottingham Vet School undergraduate we hope to model different aspects of equine wound healing and predict how long different types of wounds take to heal. Please only restrain and take photographs of your horse if it is safe to do so and your horse's temperament allows. Do not place yourself, your horse or anyone else helping to handle the horse in any unnecessary danger. If it is impractical/unsafe to take photographs please remember to still submit the online forms.

If you do not have any photos of the wound or would not like to submit them, please do not worry! It would be great if you could continue to complete the rest of the form either way.

2. Please indicate the date that you took the photographs of the healed wound

   Example: January 7, 2019

3. Photographs of the wound from a distance

   Files submitted:

4. Close up photographs of the wound

Files submitted:

Details of horse

We are aware that some of these questions have already been asked in form 1, they are repeated in subsequent forms so that researchers can match up different forms from the same horse

5. What is the name of your horse?

____

6. How old is your horse?

____

7. What gender is your horse?

Mark only one oval.

- ( ) Gelding
- ( ) Stallion
- ( ) Mare

Wound outcome

8. Date you believe the wound to have completely healed

____

Example: January 7, 2019

9. Was euthanasia required as a result of this wound?

Mark only one oval.

- Yes
- No
- Prefer not to say

10. How satisfied with you were the rate of wound healing?

Mark only one oval.

- Wound healed faster than expected
- Wound healed at the rate I expected
- Wound healed slower than expected
- Did not know what to expect

11. Has your horse/is your horse predicted to return to the same capacity of work prior to wound infliction

Mark only one oval.

- Yes, horse was never out of work
- Yes, horse has already returned to the same capacity of work
- Yes, horse is predicted to return to the same capacity of work in the future
- No, horse is not predicted to return to the same capacity of work
- Unsure
- Other:

12. How long did it take for your horse/how long is it predicted for your horse to return to the same capacity of work prior to wound infliction?

Please specify units, e.g. days/weeks/months

13. Grade any lameness caused as a result of the wound

Mark only one oval.

- Horse is sound
- Cannot see lameness at walk and it is sometimes but not always there under certain circumstances (e.g. when turning)
- Lameness is difficult to observe at walk but is always there under certain circumstances (e.g. uncomfortable when turning)
- Horse is bearing less weight on the affected limb at walk/when resting
- Horse cannot bear weight on the affected limb

14. If lameness occured because of the wound, how long did it take for the horse to become sound?

Please specify units, e.g. days/weeks/months, if horse is still lame due to the wound please specify this

15. Please score the cosmetic outcome of the wound

Mark only one oval.

- Excellent - Scar is faint or not evident
- Adequate - Scar is clearly visible but not excessively wide or large
- Unacceptable - Scar is wide, unsightly or exceedingly large

16. Please give details of how the wound affected your day to day management of the horse

E.g. box rest, out of work, wound care,

17. Please give details of any additional complications that resulted because of the wound

18. Was the treatment of the wound limited by any of the following?

Check all that apply.

- [ ] Finances
- [ ] Facilities
- [ ] Horse temperament
- [ ] Prefer not to say
- [ ] Other: ____________________

19. If you are happy to give the following information, what was the total cost of treatment for this wound?

20. Following on from this study, what resources/information would you like to be made available to horse owners on wound management and in what format would you prefer them to be delivered?

21. How did you hear about The Equine Wound Project?

Prize Draw

As a thank you for your participation and commitment to the Equine Wound Project, we invite you to enter your name into a prize draw, details of prizes can be found at https://www.bhs.org.uk/our-work/welfare/wounds-project/prizes

22. I consent to entering my name into a prize draw, drawn August 2019

Mark only one oval.

- Yes
- No

This content is neither created nor endorsed by Google.

Google Forms
